# Comparative Analysis of the Global Transcriptomic Response to Oxidative Stress of *Bacillus anthracis* *htrA*-Disrupted and Parental Wild Type Strains

**DOI:** 10.3390/microorganisms8121896

**Published:** 2020-11-30

**Authors:** Galia Zaide, Uri Elia, Inbar Cohen-Gihon, Ma’ayan Israeli, Shahar Rotem, Ofir Israeli, Sharon Ehrlich, Hila Cohen, Shirley Lazar, Adi Beth-Din, Avigdor Shafferman, Anat Zvi, Ofer Cohen, Theodor Chitlaru

**Affiliations:** Department of Biochemistry and Molecular Genetics, Israel Institute for Biological Research, Ness-Ziona 74100, Israel; galiaz@iibr.gov.il (G.Z.); urie@iibr.gov.il (U.E.); Inbarg@iibr.gov.il (I.C.-G.); maayani@iibr.gov.il (M.I.); Shaharr@iibr.gov.il (S.R.); ofiri@iibr.gov.il (O.I.); Sharone@iibr.gov.il (S.E.); Hilac@iibr.gov.il (H.C.); Shirleyl@iibr.gov.il (S.L.); Adib@iibr.gov.il (A.B.-D.); ashafferman@gmail.com (A.S.); Anatz@iibr.gov.il (A.Z.); Oferc@iibr.gov.il (O.C.)

**Keywords:** *Bacillus anthracis*, anthrax, oxidative stress, HtrA, transcriptomics, RNA-seq, stress response

## Abstract

We previously demonstrated that the HtrA (High Temperature Requirement A) protease/chaperone active in the quality control of protein synthesis, represents an important virulence determinant of *Bacillus anthracis*. Virulence attenuation of *htrA*-disrupted *Bacillus anthracis* strains was attributed to susceptibility of Δ*htrA* strains to stress insults, as evidenced by affected growth under various stress conditions. Here, we report a comparative RNA-seq transcriptomic study generating a database of differentially expressed genes in the *B. anthracis* *htrA*-disrupted and wild type parental strains under oxidative stress. The study demonstrates that, apart from protease and chaperone activities, HtrA exerts a regulatory role influencing expression of more than 1000 genes under stress. Functional analysis of groups or individual genes exhibiting strain-specific modulation, evidenced (i) massive downregulation in the Δ*htrA* and upregulation in the WT strains of various transcriptional regulators, (ii) downregulation of translation processes in the WT strain, and (iii) downregulation of metal ion binding functions and upregulation of sporulation-associated functions in the Δ*htrA* strain. These modulated functions are extensively discussed. Fifteen genes uniquely upregulated in the wild type strain were further interrogated for their modulation in response to other stress regimens. Overexpression of one of these genes, encoding for MazG (a nucleoside triphosphate pyrophosphohydrolase involved in various stress responses in other bacteria), in the Δ*htrA* strain resulted in partial alleviation of the H_2_O_2_-sensitive phenotype.

## 1. Introduction

The Gram-positive spore-forming obligate pathogen *Bacillus anthracis* represents the etiological agent of anthrax, a currently rare disease in humans, yet potentially associated with intentional bioterror use [1,2]. In the most severe respiratory form, *B. anthracis* infection is initiated by inhalation of spores which germinate into fast dividing vegetative cells which secrete toxins and virulence factors during growth in the host (for review, see in [3,4,5]), resulting in massive bacteremia and consequently generalized systemic failure and death.

The lethality of anthrax has been attributed to two main aspects of *B. anthracis* pathogenesis: the activity of the bacterial exotoxins and the remarkable proliferous nature of the bacteria in the host. This latter aspect of *B. anthracis* pathogenicity suggests that the pathogen excels in exploiting nutritional resources available in the host and is highly adapted to cope with stress constraints encountered in the course of infection. The *B. anthracis* exotoxins, encoded by genes located on the virulence plasmid pXO1, are compose of a binary combination of three proteins (Lethal Toxin (LF), Edema Toxin (ET), and Protective antigen (PA)). PA, the common subunit of both toxins, is capable of eliciting a protective immune response, and thus its administration represents the basis for all preventive anthrax countermeasures [4,6]. A second virulence plasmid, pXO2, encodes for functions required for the biosynthesis of a poly-glutamate antiphagocytic capsule necessary for survival of the bacteria in the host [7,8].

Anthrax is acknowledged as a toxinogenic disease, owing to the lethality of pure toxin preparations. Yet, during infection, *B. anthracis* secretes a large number of proteins, many of which bear biological functions indicative of a role in the onset and progression of the disease [9,10,11,12,13,14,15,16,17]. As of today, very few proteins, other than the classic toxins, have been suggested to play an essential role during *B. anthracis* infection, based on the attenuated virulence of null mutants entailing targeted disruption of specific genes. Such are the Mn transporter MntA [18], the ClpX protease [19], proteins required for siderophore biosynthesis [20], nitric oxide synthase [21], the pXO1-encoded BslA adhesin [22], the product of the *purH* gene involved in the purine biosynthesis [23], and the extracellular protease and chaperone HtrA whose role in stress response is addressed in the current study [4] (see in [13] for a list and discussion of reported *B. anthracis* attenuating mutations).

The HtrA (High Temperature Requirement A) family of serine proteases are central players in the context of protein synthesis quality control. HtrA proteins are structurally and functionally conserved across a wide range of evolutionary distinct phylogenetic classes both in prokaryotes and eukaryotes [24]. They exhibit the dual biological activities of chaperones and proteases, are involved in manifestation of virulence of many pathogens, and consequently represent potential targets for therapy [25,26,27,28,29,30]. HtrA proteases exhibit a characteristic structure, composed of an N-terminal trypsin-like serine protease domain and at least one C-terminal PDZ domain that recognizes substrates and activates the protease function [31,32]. In *E. coli* and *B. subtilis*, the HtrA family of proteases are important for the survival of the bacteria under different stress regimens [26,28,33]. In Gram-positive bacteria, the HtrA chaperones/proteases are closely associated with the SecA membrane-translocation machinery suggesting that their targets are constituted by secreted proteins [34]. HtrA was invoked as being directly involved in the proteolytic processing or secretion of specific virulence-associated proteins such as SpeB and Hemolysin in *Streptococcus pyogenes* [35,36], Pertussis toxin S1 [37], and Adhesin P1 of *Streptococcus mutans* [38]. In *Helicobacter pylori*, HtrA was shown to facilitate virulence manifestation by direct proteolysis of host proteins E-cadherin [39,40], as well as by its central role in stress resilience [41].

High-throughput genomic/proteomic/serologic surveys of *B. anthracis* (reviewed in [4,16,42]) showed that HtrA belongs to a class of exposed immunogenic putative vaccine candidates. *B. anthracis* HtrA emerged as a potential early secreted anthrax biomarker [11,12,43], and its disruption in the toxinogenic *B. anthracis* Vollum or Sterne strains resulted in a dramatic attenuation in the guinea pig, murine, and rabbit models of anthrax [13,44]. As HtrA is essential for manifestation of *B. anthracis* pathogenesis, disruption of the *htrA*-gene in the non-capsular Sterne strain served for the development of an efficacious and safe next-generation live attenuated anthrax spore vaccine [13,45]. The phenotype associated with disruption of the *htrA* gene in either virulent or non-virulent (virulence plasmid-cured) strains established that HtrA is necessary for tolerance of various stress stimuli and for modulation of several bacterial proteins potentially involved in the stress response [31,44]. Most notably, *htrA*-disrupted bacteria exhibited significant sensitivity to hydrogen peroxide-induced oxidative stress correlating with a delayed multiplication of the bacteria in a macrophage infection assay. Of note, resilience to oxidative stress in the host macrophage in the course of infection is considered to represent an important feature of pathogenic bacteria, in general, and *B. anthracis* in particular [46,47,48,49,50].

In the current report, we address the role of HtrA in the response of *B. anthracis* to hydrogen peroxide-induced oxidative stress by conducting a comparative transcriptomic study which generated a database of differentially expressed genes (DEGs) in the *B. anthracis htrA*-disrupted and wild type parental strains. Selected genes emerging from this comparative study were further interrogated for their expression modulation under additional stress treatments, enabling identification of several genes induced specifically by oxidative stress in an HtrA-dependent manner. The study substantiates the important role HtrA plays in the adaptation of the bacteria to the hostile environment, and strengthens the notion that in addition to its direct role in post-translation processing and quality control of proteins associated with its chaperone and protease catalytic activities, *B. anthracis* HtrA exerts a pleotropic effect on gene expression under oxidative stress conditions.

## 2. Materials and Methods

### 2.1. Bacterial Strains, Media, Growth Conditions and Stress Treatment

*B. anthracis* parental strain ΔVollum (acapsular and nontoxinogenic, referred in this report as wild type (WT)) and the *htrA*-disrupted strain [44] were cultured in brain–heart infusion (BHI; DIFCO/Becton Dickinson, MD, USA) media at 37 °C to mid-log phase in triplicates. Cells were then split into twin cultures, washed, diluted into fresh media and grown in BHI in the presence or absence of 3mM or 5 mM H_2_O_2,_ 4% NaCl, or at 41 °C. Optical density (OD at a wavelength of 660 nm) was recorded at several time points in order to determine the growth rate of the two strains under different stress conditions. In the case of H_2_O_2_ treatment, samples (4 mL) generated from each culture (WT or Δ*htrA)* were collected from each culture before treatment and 10 min post-stress initiation (in the presence of absence of H_2_O_2_), centrifuged and pellets were transferred to −70 °C for further use. In the case of heat and salt stress, no samples were collected before stress treatment.

### 2.2. RNA Isolation, Reverse Transcription, and Real-Time PCR Analyses

Primers employed for the RT-PCR analyses are listed in Supplementary Table S1. Total RNA was extracted from bacteria samples using the RiboPure-Bacteria kit (Ambion, TX, USA) according to the manufacturer’s instructions. Genomic DNA was removed by adding DNase buffer (at a ratio of 1:9) and 4 µL of DNase I was added to the samples, followed by incubation at 37 °C for 30 min. DNase inactivation reagent was subsequently added at a 1:5 (*v*/*v*) ratio, followed by incubation at room temperature for 2 min. Samples were then centrifuged (1 min, 13,000× *g*) to pellet the DNase inactivation reagent and the supernatant (RNA) was transferred into a fresh collection tube and kept at −70 °C until use. Complementary DNA was generated using the Reverse Transcription System (Promega, WI, USA). Random hexamers (0.75 µL) with 0.25 µL of oligo dTs were added to 0.5 µg of RNA. The mix was heated (70 °C, 5 min); cooled to 4 °C and supplemented with 4µL MgCl_2_, 2 µL reverse transcription buffer, 2 µL dNTPs, 0.5 μL recombinant RNasin ribonuclease inhibitor, and 0.65 µL AMV reverse transcriptase. The reaction mixture was incubated at 37 °C for 10 min, heated to 40 °C for 10 min, 42 °C for 60 min, and cooled to 4 °C. The resulting cDNA was diluted 1:5 and amplified using the PerfeCTa SYBR Green Supermix kit (Quanta BioSciences, MA, USA) with 500 nM gene-specific primers. Experiments were performed using the 7500 ABI Real-Time PCR system (Applied Biosystems, MA, USA). The constitutively expressed *gatB* gene was used as internal control [51]. Plasmid DNA containing the analyzed gene served as a template for cDNA quantification. In all cases, mock reverse transcriptase reactions, served for determining possible chromosomal DNA contamination, resulted in <5% of the total copies measured.

### 2.3. RNA Sequencing, Transcriptome Assembly and Differential Expression Analysis

The RNA samples obtained from each of the duplicated or triplicated growth conditions were sequenced in-house (IIBR, Ness Ziona, Israel) using an Illumina Genome Analyzer IIe system (Illumina, CA, USA) with TruSeq sequencing-by-synthesis (SBS) kit version 2 reagents. Raw data for each sample were analyzed for QC using FastQC (https://www.bioinformatics.babraham.ac.uk/projects/fastqc). Raw sequence reads were mapped to the B. anthracis Ames Ancestor reference genome (GenBank accession number NC_007530) using Novoalign, version 3.02.07 (http://www.novocraft.com/). The raw count per gene was calculated using HTSeq [52], version 0.6. In each set of reads, at least 90% was mapped to the reference genome. Reads not mapped were excluded from further analysis. Differential expression analyses of the genes under various conditions were performed using the R package DESeq version 1.16.0 [53]. The reproducibility of the biological replicates for all the conditions examined was assessed by Principal Component Analysis (PCA) using the R package DESeq2 [54]. Similarity matrix and hierarchical clustering were generated using the R packages gplots and RColorBrewer. Default settings in all analytical software programs were used. The transcriptomic data have been deposited to the NCBI database [55]. GEO (Gene Expression Omnibus) accession of the entire transcriptome series: GSE151208. SRA Bioproject: PRJNA635127. GEO title of project: Transcriptome RNA Sequencing Data Sets of *B. anthracis* Vollum Δ*htrA* and Parental Isogenic Wild type strains under Oxidative Stress Conditions. SRA and GEO accession numbers of individual RNA data sets (see Section 3.2): Sample 1a: SAMN15018196; GSM4568574. Sample 1b: SAMN15018194; GSM4568575. Sample 2a: SAMN15018192; GSM4568576. Sample 2b: SAMN15018191; GSM4568577. Sample 3a: SAMN15018189; GSM4568578. Sample 3b: SAMN15018187; GSM4568579. Sample 4a: SAMN15018185; GSM4568580. Sample 4b: SAMN15018181; GSM4568581. Sample 4c: SAMN15018200; GSM4568582. Sample 5a: SAMN15018198; GSM4568583. Sample 5b: SAMN150181203; GSM4568584. Sample 6a: SAMN15018205; GSM4568585. Sample 6b: SAMN15018202; GSM4568586. Sample 4c: SAMN15018201; GSM4568587. The expression levels used for classification of DEGs were obtained by averaging those measured in duplicates or triplicates representing same strain and same growth condition. DEGs (adjusted *p* value < 0.01) that exhibited a modified pattern of expression in both strains, those that were uniquely modulated in the WT, and those that were uniquely modulated in the Δ*htrA* strains are documented in Supplementary Tables S2–S4, respectively. Supplementary Tables S3 and S4 are each divided into separate sheets for upregulated and downregulated genes.

### 2.4. Genome Annotations and Gene Ontology Analysis

The *B. anthracis* Ames ancestor genome sequence (GCA_000008445.1) was obtained from the NCBI database. The genome was annotated based on protein homology to the “Bacteria” phylogenetic domain using the OmicsBox software BlastX tool (https://www.biobam.com/omicsbox) with default parameters. Functional classification of DEGs with adjusted *p* value < 0.05 that were uniquely modulated in either the WT or the Δ*htrA* strains was carried out using the OmicsBox mapping tool [56], providing a global view of GO terms for the genes. Fisher’s exact test for these DEGs was performed using the OmicsBox software to estimate the association between the DEG and specific GO categories when compared with the background genes. Throughout the article, individual genes are referred by their GBAA NCBI locus tag numbers for the *B. anthracis* Ames chromosome.

### 2.5. Overexpression of MazG in the ΔVollum ΔhtrA Strain and Evaluation of Its Involvement in Oxidative Stress Response

The gene encoding MazG was amplified by PCR from the *B. anthracis* ΔVollum strain using the MazG forward (AGACCTAGATCTTATACAAAAAGGAGTACGTATATGAATC-AAAATACAATAAA) and the MazG reverse (AGATAGACCTGGATCCCTATAGT-TCGTTATATTTTTTAT) primers. The product (as a SnaBI-BamHI fragment) was ligated into the linearized pASC-α *Bacillus* expression vector (Ap^R^Cm^R^, pC194 ori [14,44]), removing the *pagA* gene resulting in the MazG expression vector pASC-MazG. Thus, the MazG coding sequence replaced the *pagA* gene downstream of the *pagA* ribosome binding site and the α-amylase promoter. The plasmid pASC-MazG was used to transform the methylation-deficient *E. coli* strain GM2929 as previously described [44], after which it was introduced into a competent ΔVollum Δ*htrA* strain. Recombinant colonies were plated on LB medium containing 7.5 µg/mL chloramphenicol. The *B. anthracis* strains Δ*htrA* or Δ*htrA/MazG* (overexpressing the gene encoding for MazG) were cultured in BHI media at 37 °C for 2.5–3 h. Cells were then split into BHI cultures containing 0, 1, 3, 5, or 10 mM H_2_O_2_ and allowed to grow for 150 min. OD was determined at several time points as described.

## 3. Results and Discussion

### 3.1. Expression of Selected Oxidative Stress Response Genes in Response to H_2_O_2_

**Figure 1:**
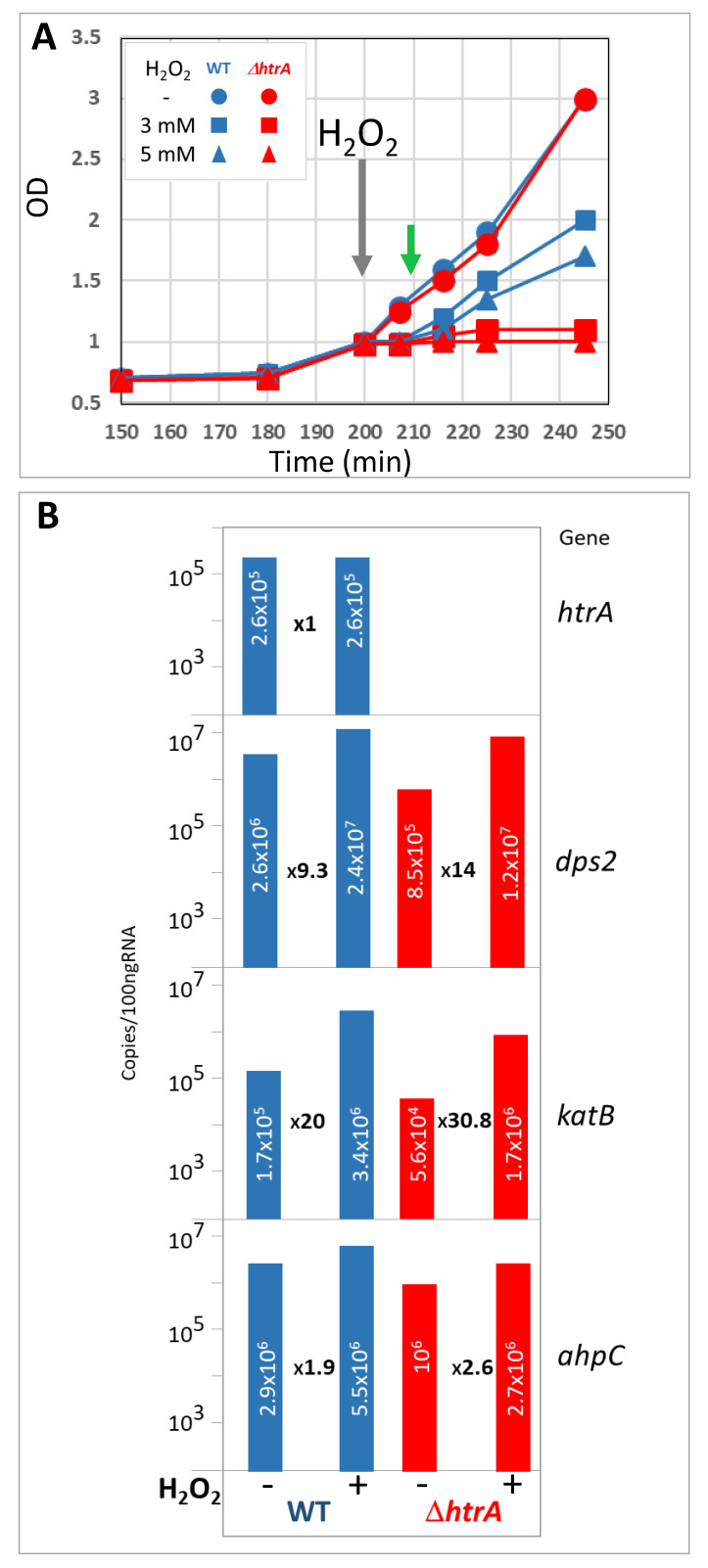
Hydrogen peroxide effect on the growth profiles and expression of selected genes of *B. anthracis* WT and Δ*htrA* strains. (**A**) Hydrogen peroxide effect on the growth profiles of *B. anthracis* WT and Δ*htrA* strains. The two strains were grown in BHI media for 3 h, then split into twin cultures and treated with 3 mM or 5 mM H_2_O_2_, as indicated (see inset legend). The gray arrow indicates the time point after addition of H_2_O_2_ at cell densities of OD600 = 1. Full growth curves of various strains of *B. anthracis* were previously extensively documented [13,44,45,57]. (**B**) Hydrogen peroxide effect on expression of selected genes belonging to the oxidative stress regulon. RNA was prepared from samples collected 10 min after onset of H_2_O_2_ treatment (see green arrow in panel A). Transcription levels of the genes *htrA* (GBAA3660), *dps2* (GBAA5290), *katB* (GBAA1159), and *ahpC* (GBAA0345) were determined by qRT-PCR. The measured values (expressed as copies/100 ngRNA) are indicated within the histograms. The fold increase in the abundance of each transcript upon H_2_O_2_ treatment is indicated between the histograms. The data depicted were obtained in one representative experiment, of three independent iterations, which yielded similar results (<10% variability).

We have previously shown that *B. anthracis* tolerance to hydrogen peroxide (as well as to other stress regimens) is significantly affected by *htrA* gene disruption [13,31,44]. Based on this observation, it was postulated that the significant virulence attenuation exhibited by the fully virulent Vollum or the acapsular Sterne strains upon abrogation of HtrA expression, may be attributed to inability to respond optimally to oxidative stress. The data depicted in Figure 1 confirmed that *htrA* disruption in the non-toxinogenic non-encapsulated (devoid of the pXO1 and pXO2 virulence plasmids) *B. anthracis* Vollum strain impacted its ability to tolerate oxidative stress induced by increasing concentrations of H_2_O_2_. The growth rate of both the WT and the Δ*htrA* strain under stress-free conditions was identical; however, addition of H_2_O_2_ resulted in an immediate growth arrest in both strains. However, while the growth of the WT cells exhibited a short lag period after which it restored its initial rate, the growth of the Δ*htrA* strain was irreversibly blocked in the presence of H_2_O_2_. These results are in line with our previously published results [13,44,57].

Previous studies of the response of *Bacillus subtilis* and *B. anthracis* to stress have shown that H_2_O_2_ treatment induces the expression of several genes attributed with a major role in mediating the resilience of the cells to oxidative stress [58,59,60]. Therefore, we verified whether the susceptibility of the Δ*htrA* strain to hydrogen peroxide may involve low expression levels of such genes. The expression pattern following H_2_O_2_ treatment of the following three selected genes, known to play a major role in the resilience to oxidative stress, was therefore determined by RT-PCR analysis: *katB* (encoding the major catalase of the bacteria, involved in the peroxide neutralization [60]), *dps2* (whose product contributes to peroxide stress resistance by protecting DNA from oxidative damage and sequestering cellular free iron to prevent the production of hydroxyl radicals [61,62]), and *ahpC* (encoding for the active subunit of the enzyme alkyl hydroperoxide reductase, known to be essential under oxidative stress conditions [63]). We observed that the transcription of all three genes was induced rapidly upon treatment with hydrogen peroxide in both strains, exhibiting similar induction indexes (Figure 1B). For example, expression of *katB* gene, the main catalase protecting *B. anthracis* against peroxide stress, increased >20 fold in WT and >30 fold in the Δ*htrA* strain. These results suggested that the enhanced sensitivity of the Δ*htrA* mutant to H_2_O_2_ does not involve the activity of the major players in the response to hydrogen peroxide. Furthermore, the expression level of *htrA* itself remained unaffected in the WT strain upon peroxide treatment (Figure 1B). Consequently, it may be hypothesized that the increased sensitivity of the mutated strain to peroxide involves a more pleiotropic role of HtrA. Such an effect may indicate that HtrA acts directly or indirectly also as a regulatory factor necessary for the stress response, in addition to its major catalytic activity—proteolysis of misfolded proteins accumulating under stress conditions. Indeed, a study of the phenotype of *B. anthracis* cells expressing various mutated forms of HtrA showed that engineered forms of HtrA, deprived of the proteolytic catalytic activity, were efficient in promoting upregulation of the starvation-induced NprA extracellular protease [31]. NprA is one of the most abundant proteins secreted by the WT strain under low-nutrient conditions, yet undetected in the secretome of *B. anthracis* Δ*htrA* strains [31,44,64]. We therefore concluded that understanding the virulence-related role of HtrA in general and its activity in the stress response in particular, may benefit from a comparative global examination of the pattern of expression of additional genes under oxidative stress in the HtrA-disrupted and WT strains.

### 3.2. Overview of the Transcriptome Analysis in Response to Oxidative Stress

**Figure 2:**
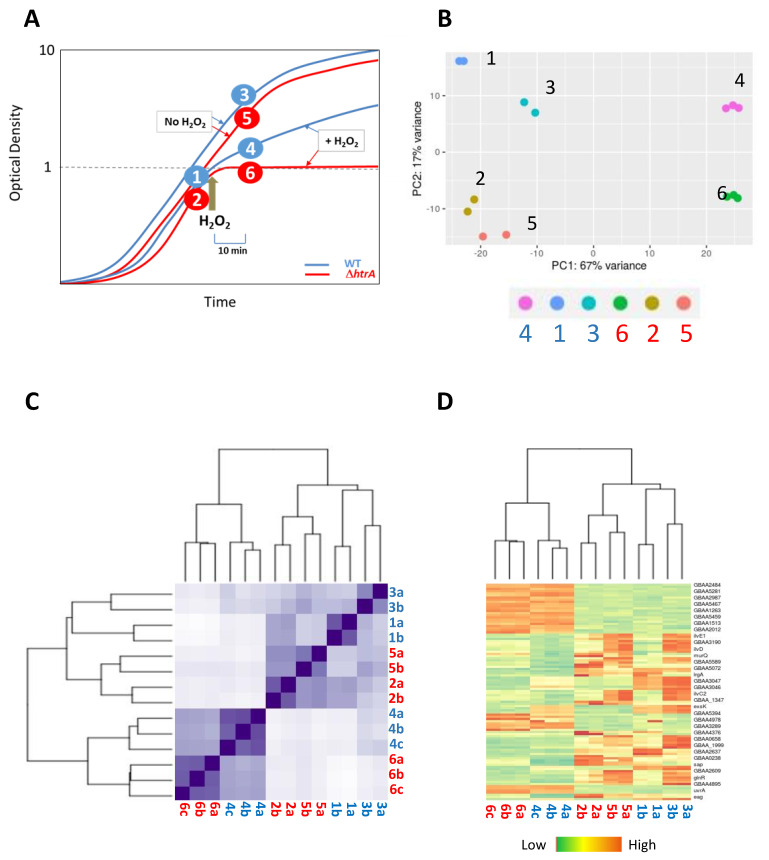
(**A**) Schematic representation of the cultures which served for collecting the WT and Δ*htrA* samples for transcriptomic analysis. All WT samples (samples 1, 3, and 4) as well as the growth curve are in blue while those of the Δ*htrA* strain (samples 2, 5, and 6) are in red. Samples 1 and 2 were collected before dividing each culture into tween flasks and continuing growth in the presence (samples 4 and 6) or absence (samples 3 and 5) of 3 mM H_2_O_2_. Samples 3–6 were collected 10 min after the onset of H_2_O_2_ treatment. Samples 1, 2, 3, and 5 were done in duplicates (marked a and b, see below), and samples 4 and 6 were done in triplicates (marked a, b, and c, see below). The RNA seq transcriptomic data of all samples were deposited to the NCBI database (as detailed in [55]). (**B**) Principal component analysis (PCA; after regularized log transformation) of gene expression of the various transcriptomes indicated in panel A. The dots represent distinct replicates (duplicates for samples 1, 2, 3, and 5, and triplicates for samples 4 and 6); their clustering indicating high similarity, underlines the reproducibility of the transcriptomic data. (**C**) Correlation heatmap of Euclidean sample distances after regularized log transformation. The dendrograms on the left and upper sides indicate hierarchical clustering of the duplicate and triplicate samples. Note also the similarity between transcriptomes 1 and 3 and between transcriptomes 2 and 5, which represent cultures grown in the absence of H_2_O_2_, as indicated in panel 1. (**D**) Heatmap diagram of the relative expression level of the 100 genes with the highest variance across samples. Similar to panel C, the dendrogram at the top indicate hierarchical clustering of the duplicate and triplicate samples as well as the similarity between the H_2_O_2_ treated and untreated-culture transcriptomes in each of the 2 strains.

For the RNA-seq comparative transcriptome analysis, the Δ*htrA* and WT strains were grown in biological triplicates or duplicates and RNA was prepared from the following samples (as schematically described in Figure 2A): samples 1 and 2, collected from the initial cultures before treatment, representing the WT and Δ*htrA* strains, respectively, in duplicates. Samples 4 and 6, in triplicates, collected 10 min post-treatment with H_2_O_2_, from the WT and Δ*htrA* strain, respectively. Samples 3 (WT) and 5 (Δ*htrA*), control untreated (without H_2_O_2_) groups in duplicates, handled similarly to samples 4 and 6. Transcriptomic quantification of RNA representing duplicates generated highly similar gene expression data, as demonstrated by the principal component analysis (PCA, Figure 2B), heat map of Euclidean sample distances (R2 value correlation between samples, Figure 2C), and heat map of the 100 genes with the highest variance across samples (Figure 2D). These evaluations established the high reproducibility of the transcriptome RNA-seq analysis and consistency of the results in the independent duplicated or triplicated experimental groups. The expression data of each particular gene from duplicated or triplicated experimental groups were averaged prior to the analysis. The transcriptomic data have been deposited to the NCBI database [55]. The transcriptomes determined in the absence of H_2_O_2_ (i.e., samples 1 and 3 for the WT strain, and 2 and 5 for the Δ*htrA* strain, see Figure 2A) were highly similar. Accordingly, the subsequent analysis of the differentially expressed genes (DEGs) was based on comparison of the H_2_O_2_ regulon of the WT strain (determined by comparing sample 3 to 4) to that of the Δ*htrA* strain (determined by comparing sample 5 to 6).

**Figure 3:**
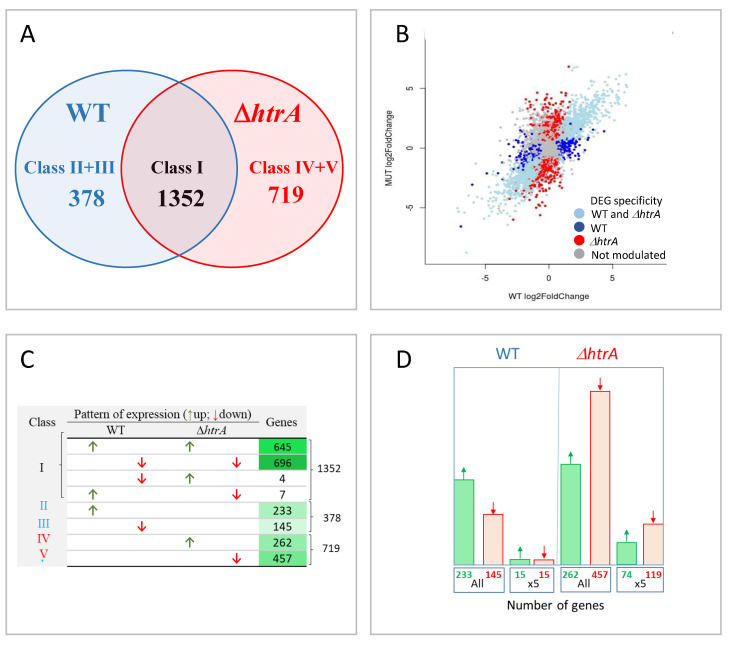
(**A**) Venn diagram of the three major categories of DEGs. Number of genes whose pattern of expression is modified by the H_2_O_2_ treatment in both strains (Class I), in the WT strain (classes II and III), or in the Δ*htrA* strain (Classes IV and V) is indicated. (**B**) All genes expression pattern scatterplot of log2 fold change. Each dot represents an individual gene; its x value is the log2 fold-change in the WT, and its y values is the log2 fold-change in the *ΔhtrA* strains, before and after H_2_O_2_ treatment. Dot color significance: gray dots represent genes exhibiting a pattern of expression not modified by stress; blue dots represent genes modulated in the WT strain only; red dots represent genes modulated in the Δ*htrA* strain only; light blue dots represent genes modulated in both strains. Note the diagonal trend of the scattered dots in the latter category (light blue) indicating that these genes exhibited a similar pattern of modulation in both strains. (**C**) Number of genes in each of the 5 Classes of DEGs. Upregulation and downregulation of genes are indicated by green ascending arrows or red descending arrows, respectively. Note that Class I genes comprise all the genes which exhibited a modified pattern of expression in both strains, both upregulated and downregulated; the vast majority of the genes in this class exhibited either upregulation or downregulation in both strains, and only a minority exhibited an inverted pattern. A green color gradient was applied to the table-cells in the right column according to the number of genes in each class. (**D**) Histograms representing the number of Class II and III (left panel, WT-specific modulated genes) and classes IV and V (right panel, Δ*htrA*-specific modulated genes), as calculated by considering all the significantly modulated (indicated as “all”) or by a more stringent 5-fold modulation index (indicated as “×5”). An adjusted *p* value of < 0.01 was applied. Histograms of upregulated genes are green, those representing downregulated genes are red.

Quantification of the number of DEGs exhibiting transcription affected by the H_2_O_2_ treatment (Figure 3) indicated that oxidative stress significantly modulated the expression of 2449 genes which could be further categorized into 5 major classes: Class I genes that were induced or repressed upon treatment in both strains (1352 genes, listed in Supplementary Table S2), Class II genes that were uniquely upregulated in the WT strain (233 genes, Supplementary Table S3 sheet 1), Class III genes that were uniquely downregulated in the WT strain (145 genes, Supplementary Table S3 sheet 2), Class IV genes that were uniquely upregulated in the *ΔhtrA* strain (262 genes, Supplementary Table S4 sheet 1), and Class V genes that were uniquely downregulated in the Δ*htrA* strain (457 genes, Supplementary Table S4 sheet 2).

The vast majority of the 1352 genes that were significantly modulated in both strains (Class I) exhibited a similar expression trend (i.e., were upregulated or downregulated in both the *WT* and the Δ*htrA* strains, Figure 3B,C). This result suggested that these genes were differentially expressed in response to H_2_O_2_ in an HtrA-independent manner. Only a minority (11 out of the 1352 Class I genes) exhibited an opposite expression trend. Four out of these 11 genes were upregulated in the Δ*htrA* strain and downregulated in the WT strain. These genes may be involved in “compensation” processes occurring in the Δ*htrA* strain for surviving the stress in the absence of HtrA. Seven genes exhibited opposite modulation, i.e., upregulated in the WT and downregulated in the Δ*htrA* strain. For example, the GBA3848 gene, encoding a DeoR family transcription regulator, was upregulated in the WT while downregulated 3-fold in the *htrA*-disrupted strains (see expression levels in Supplementary Table S2). Proteins belonging the DeoR family act as repressors of the *deo* operon which encodes enzymes that are involved in nucleoside catabolism [65]. Upregulation of DeoR is therefore consistent with a lower level of DNA replication associated with the stress-induced transient growth stagnation possibly necessary for the response to oxidative stress in the in the WT strain. Of note, other DeoR regulatory proteins also exhibited downregulation specifically in the *htrA*-disrupted (while their transcription level was not modified in the WT strain) and belong consequently to the Class V genes (Figure 3C, see GO-enrichment analysis below). Since the current transcriptomic analysis focused on the HtrA-related stress response (i.e., inspection of genes specifically up or down regulated in one of the strains only), the Class I DEGs (modulated in both strains) was not further inspected.

Notably, a significantly larger number of genes were modulated in the *htrA*-disrupted strain compared to the WT strain following H_2_O_2_ treatment (719 genes compared to 378 genes, Figure 3A,C,D), in line with the concept that the Δ*htrA* strain was much more affected by oxidative stress than the parental strain. Furthermore, while in the WT strain there were more upregulated than downregulated genes (233 class II DEGs compared to 145 Class III DEGs), in the case of the Δ*htrA* strain, a considerable higher proportion of genes were downregulated (compare Class V to Class IV, Figure 3C,D). Conceivably, this observation reflects the fact that a large number of genes, not essential for growth, were silenced in the *htrA* disrupted strain, which is more severely affected by the H_2_O_2_ treatment. The observation that a high number of genes were modulated, and in particular downregulated, only in the Δ*htrA* strain, was confirmed also by the subsequent functional analysis (see below).

### 3.3. Determination of Functional Categories of DEGs by Gene Ontology (GO) Analysis

**Figure 4:**
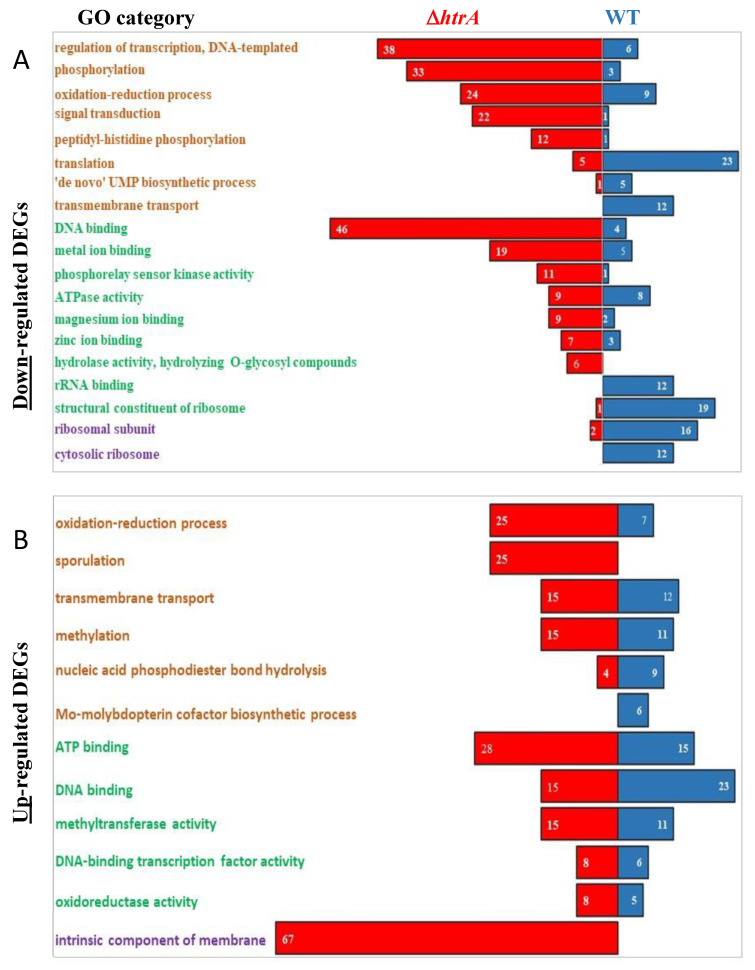
Gene Ontology (GO) analysis of downregulated (**A**) and upregulated (**B**) DEGs upon H_2_O_2_. (**A**). Inherent to the GO enrichment analysis, one particular gene may belong to more than one GO category. For the GO analysis, an adjusted *p* value of < 0.05 was applied.

The standardized gene function classification system Gene Ontology (GO) was implemented for exploring the physiological significance of the DEGs [66,67,68]. Accordingly, GO assignments were used to classify *B. anthracis* genes that were differentially expressed upon oxidative stress into functional groups based on the common biological roles of their products. For an updated version of the *B. anthracis* ORF functional annotation, the genome sequence data of the reference *B. anthracis* Ames ancestor strain (NCBI accession GCA_000008445.1) was subjected to BLAST analysis using the OmicsBox package. The resulting updated gene functional annotations were subsequently subjected to GO analysis which enabled classification of genes that were uniquely modulated in either the WT or the Δ*htrA* strain, based on their putative biological and/or catalytic role. This analysis (referred to as “GO enrichment analysis”, summarized in Figure 4) resulted in assignment of a particular gene to one or more functional categories (referred as GO terms, according to the GO classification), which belong to one of the relevant GO functional divisions (or roots): biological processes, molecular functions, or cellular components. DEGs exhibiting a statistically significant change in their transcriptional level (adjusted *p* value < 0.05) from each of the 4 classes of genes specifically modulated only in one of the strains (Classes II–V, see Figure 3A,C) were subjected to GO enrichment analysis (see Figure 4A,B, which summarizes the GO analysis of the downregulated and upregulated DEGs, respectively).

Several general conclusions could be drawn from inspection of the number of genes in each GO category (Figure 4): (i) a significantly higher number of genes were modulated upon H_2_O_2_ treatment in the Δ*htrA* strain. This was true both for down- and upregulated genes, but was most pronounced in the case of downregulated genes (Figure 4A), in which almost all categories included more genes uniquely modulated in the Δ*htrA* strain (see detailed reference to individual groups and genes, below). (ii) The majority of categories of upregulated DEGs were more enriched in the Δ*htrA* than in WT the strain, in terms of the respective number of genes (Figure 4B). It is important to point out that DEGs which were upregulated solely in the WT strain (compiled in Supplementary Table S3, sheet 1) represented a category of choice for identification of genes potentially contributing to the failure of the Δ*htrA* strain to properly respond to stress; therefore, this category was further addressed in the detailed analysis below. (iii) A large number of WT downregulated genes are involved in translation and ribosome structure (Figure 4A, and see discussion below). (iv) Strikingly, the term “intrinsic component of membrane” was overrepresented among the enriched terms of the Δ*htrA* upregulated genes (Figure 4B). This phenomenon may be a mere manifestation of the fact that in bacteria many metabolic activities are carried out by proteins localized in membranes. Yet, it is tempting to assume that in the context of the response to stress (which is impaired in the Δ*htrA* strain), many membrane-localized proteins were more rapidly upregulated than those residing in other components of the cell, since the membrane represents the interphase between the bacteria and the surroundings. As we attempted to concentrate only on the function of the DEGs, their attribution to GO terms belonging to the “cellular component” division (which solely indicated the localization of the gene products), was beyond the scope of the current study and therefore not further datamined. We note also that all the genes belonging to the “intrinsic component of membrane” GO category were also attributed to other GO terms; therefore, their exclusion from the current analysis did not result in substantial misrepresentation of the functions impacted by oxidative stress.

### 3.4. Downregulation of Transcriptional Regulators in the ΔhtrA Strain Under Oxidative Stress

Inspection of individual DEGs in the various functional groups (Figure 4A) revealed a significant difference between the WT and the Δ*htrA* strains with respect to the downregulation of genes involved in transcription regulation. Thus, 71 unique genes involved in transcription regulation activities (included in “regulation of transcription, DNA-templated” and “DNA binding” GO terms) were downregulated in the Δ*htrA* strain, while only 10 unique genes belonging to the same groups were downregulated in the WT strain. The MarR, DeoR, RocR, TetR, and PadR families of transcription regulators were amongst the genes exhibiting a transcriptional level downregulated by a factor of over 5-fold in the Δ*htrA* strain. These regulators are involved in the adaptive response to various stress conditions as well as virulence in many bacterial strains including *B. anthracis*. More specifically, five members of the MarR family transcription regulators were downregulated uniquely in the Δ*htrA* strain. Two out of the five genes (GBAA0662 and GBAA5284) were downregulated 10- and 9-fold, respectively. MarR transcriptional regulators are known to play roles in ligand-mediated regulation of virulence factor production and bacterial responses to environment stress [69]. Members of this family are reportedly critical to the virulence of a variety of bacterial pathogens, including *Salmonella typhimurium*, *Yersinia enterocolitica*, *Vibrio cholera*, and *Staphylococcus aureus* [70]. The putative *B. anthracis* MarR family transcriptional regulator GBAA1941 was downregulated by a factor of 3 in the Δ*htrA* strain. Most notably, this observation confirms an early global transcriptomic study of *B. anthracis* addressing genes essential for the survival of the bacteria in the host macrophages [71]. This study established that the regulator encoded by the GBAA1941 gene was upregulated upon infection of macrophages, and consequently its disruption influenced manifestation of virulence in a mouse model of inhalational anthrax. Furthermore, taken together, the data were in agreement with our previous observation that Δ*htrA* bacteria are affected in their ability to propagate in macrophage cultures [44,45]. Another group of transcriptional regulators exhibiting low transcriptional levels in the Δ*htrA* strain belongs to the DeoR family. These proteins act as repressors of the *deo* operon which encodes enzymes that are involved in nucleoside catabolism [65]. In *B. subtilis*, a DeoR regulatory protein acts as a repressor of the *dra-nupC-pdo* operon, encoding three enzymes required for deoxyribonucleoside and deoxyribose utilization [72]. The gene encoding the arginine utilization regulatory protein RocR (GBAA0489) exhibited 11-fold downregulation upon exposure to H_2_O_2_ in the Δ*htrA* strain. In *B. subtilis*, RocR acts as a transcriptional activator of the two operons, *rocABC* and *rocDEF*, that are involved in arginine and ornithine utilization, respectively [73]. Downregulation of these operons in the *htrA*-disrupted strain was therefore compatible with the general slowdown of catabolic non-essential processes under oxidative stress conditions. The TetR family transcription regulators acts as repressors of genes and operons relating to osmotic stress, biosynthesis of antibiotic and efflux pumps [74,75,76]. In addition, this family of proteins were also shown to regulate transcription of genes involved in virulence in *Vibrio cholera* [77,78,79,80], *Enterococcus faecalis* [81], and *Bacillus cereus* [82]. Accordingly, it is conceivable that genes relieved from TetR repression are necessary for survival of the *htrA*-disrupted strain under stress conditions, but not the WT strain which is more resilient to stress. The PadR family consists of a large group of transcription factors that play key roles in the regulation of biological processes underlying survival strategies such as drug resistance, antibiotic synthesis, and detoxification. For example, in *Bacillus subtilis* PadR regulates transcription in response to toxic coumaric acid [83]. *Corynebacterium glutamicum* expresses VanR (a member of the PadR family of regulators) to utilize vanillate when other carbon sources are scarce [84]. Altogether, the large number of transcriptional factors downregulated in the Δ*htrA* strain upon oxidative stress was consistent with the impaired adaptation to the stress conditions in the absence of HtrA. Future studies will determine if this phenomenon is a cause or a consequence of the hypersensitivity of this strain to stress.

Apart from the modulation of transcriptional factors, as described above, we noted that the group of DEGs specifically downregulated upon H_2_O_2_ treatment only in the Δ*htrA* strain (*p* < 0.05) included seven genes (GBAA0831, GBAA4879, GBAA4880, GBAA4374, GBAA4375, GBAA4376, and GBAA5261) that were previously reported to be highly upregulated in *B. anthracis* grown in blood [85]. These genes encode functions involved in the consumption of amino acids, possibly essential for the survival of the bacteria in the host circulation. Therefore, the observation that these genes were not active in the *htrA*-disrupted strain was in line with the notion that this strain was significantly affected in its ability to adapt to stressful conditions encountered in the host.

### 3.5. Downregulation of Translation Processes Exhibited by the WT Strain

A notable phenomenon emerging from the comparative analysis of the response to oxidative stress is the relatively large number of downregulated genes encoding for ribosomal proteins, in the WT strain, as indicated by the pattern of expression of genes belonging to the GO categories “translation”, “rRNA binding”, “structural constituent of ribosome”, “ribosomal subunit”, and “cytosolic ribosome” (Figure 4A). While the WT strain exhibited significant downregulation of 23 individual genes (which constitute these 5 GO categories, note that a gene may belong to more than one GO functional category), in the *htrA*-disrupted strain expression of only eight genes belonging to these GO categories were observed (Figure 4A). Downregulation of genes encoding ribosomal components may be necessary for limiting toxic translation errors and thereby provide a rationale for active reduction of the level of translation. This concept is supported by the reduction in the abundance of ribosomes in response to stress in *B. subtilis* [86].

### 3.6. Downregulation of Genes Encoding Metal Ion Binding Proteins

Thirty-five unique genes downregulated in the Δ*htrA* strain were attributed to three categories related to metal ion binding (metal ion binding, zinc ion binding, and magnesium ion binding), while only 10 genes classified to the same categories were downregulated in the WT strain. Yet, only the gene *alsS* (GBAA0866) encoding acetolactate synthase (AlsS) was downregulated over 5-fold, while the remaining genes exhibited only a moderate lowered transcriptional level. Of note, AlsS was shown to be associated with induction of stress response in *E. coli* [87] and to be upregulated under stress conditions in *Staphylococcus aureus* [88]. Therefore, its downregulation in the absence of HtrA may represent a manifestation of the failure of the Δ*htrA* strain to respond to oxidative stress. Interestingly, the two genes—GBAA2633 and GBAA4478 (belonging to the “metal ion binding” GO category)—downregulated in the WT strain, encode a cysteine dioxygenase and a 5-methyltetrahydrofolate-homocysteine methyltransferase, respectively (Figure 4A). Cysteine dioxygenase (GBAA2633), which is responsible for the first major step in the metabolism of cysteine, is downregulated 26-fold upon induction of oxidative stress. The second gene, homocysteine methyl transferase (GBAA4478), downregulated more than 3-fold, participates in the process of converting homocysteine to methionine. The downregulation of these two genes that are part of the biosynthesis of cysteine and methionine may imply a metabolic shift that takes place as a countermeasure against oxidative stress in the WT strain. A similar phenomenon was also described in *Caulobacter crescentus*, in which the main physiological response to oxidative stress was suggested to be represented by a shift in amino acid metabolism resulting in enhancement of the synthesis of histidine at the expense of a reduction of the cysteine and methionine synthesis [89]. Another metal ion binding protein and oxidoreductase, specifically downregulated in the Δ*htrA* strain, is Precorrin dehydrogenase, encoded by the GBAA2142 gene, a paralog of the *sirC* gene in other bacilli [90]. Oxidoreductases similar to GBAA2142 were shown to be beneficial to resilience to oxidative stress in the pathogen *Mycobacterium tuberculosis* [91,92].

### 3.7. Genes Transcriptionally Upregulated upon H_2_O_2_ Treatment

The GO enrichment gene analysis did not reveal significant differences between the WT and the Δ*htrA* strain in the number of upregulated DEGs comprised in the major GO categories. Yet, 25 DEGs belonging to the “oxidation reduction process” GO functional term were specifically upregulated in the Δ*htrA* strain, 10 of which exhibiting over 5-fold increase upon H_2_O_2_ treatment. On the other hand, this functional GO category includes only seven DEGs in the WT strain, among which only one exhibiting an increase greater than five-fold (Figure 4B and Supplementary Table S3). Two WT genes included in this category are encoding for the Spx transcription regulators, *spxA1* (GBAA1200) and *spxA2* (GBAA3456). These genes were upregulated 4- and 8-fold, respectively. Spx controls a regulon of more than 100 genes and has a key role in enabling the microorganism to cope with disulfide stress. The Spx regulators of *Bacillus subtilis* affect the expression of a large regulon in response to proteotoxic conditions, such as heat and disulfide, as well as cell wall stress [93]. Most notably, the observation that these genes fail to be upregulated in the Δ*htrA* strain under oxidative stress, are in excellent agreement with a previous report demonstrating that *B. anthracis* cells in which the expression of spxA1 was abrogated, were sensitive to diamide and hydrogen peroxide, and the *spxA1* and *spxA2* double mutant cells were hypersensitive to the thiol-specific oxidant, diamide [94].

The GO functional term “DNA binding” includes 23 genes that are upregulated in the WT (Figure 4B and Supplementary Table S3). The majority of the WT upregulated genes that belong to this GO “functional term” encode various regulatory factors, among which are the redox-sensing transcriptional regulator RexR (GBAA0263) [95]; the NrdR repressor (GBAA4824), which negatively regulates the transcription of the *nrdEF* operon, responsible for the synthesis of dNDPs necessary for DNA replication and repair [96], ArsR (GBAA3103); a metal-resistant protein [97]; the molybdopterin biosynthesis proteins (GBAA4973 and GBAA4976) involved in catalysis of a wide range of oxidation–reduction processes [98]; and TetR paralogs (GBAA4721, GBAA0955, GBAA2543, and GBAA3487). TetR proteins are involved in homeostasis, in the response to osmotic stress [74,75,76]. It is interesting to note that other members of the TetR family were found to be downregulated specifically in the Δ*htrA* strain.

Within the category of upregulated DEGs in the Δ*htrA* strain, two genes encode flavodoxin proteins (GBAA3596 and GBAA1394, belonging to the oxidation-reduction processes category, Figure 4B), whose expression increased 106- and 26-fold, respectively. Flavodoxins (Flds) are small antioxidant soluble electron transfer flavoproteins found in a wide range of bacteria [99,100,101]. Flavodoxin is typically induced as an adaptive resource under environmental or nutritional hardships allowing survival and reproduction of many organisms under deleterious conditions [101]. GBAA3596 was also reported to be significantly upregulated in *B. anthracis* grown in mammalian blood. It is conceivable that in the case of growth under oxidative stress, as described in the current study, expression of this gene represents a compensation mechanism activated only in the Δ*htrA* strain which is severely affected by the H_2_O_2_ treatment [85].

Most notably, the GO functional term “sporulation” included 25 genes uniquely upregulated in the Δ*htrA* strain and no genes in the WT strain (Figure 4B). The adjacent genes *spoVFA* (GBAA3939) and *spoVFB* (GBAA3938) belonging to this term, encoding dipicolinate synthase subunit A and B [102], were upregulated 6- and 8-fold, respectively, in the Δ*htrA* strain. These genes were reported to be expressed in *B. subtilis* during sporulation [103,104]. Three additional sporulation-related genes (GBAA4042, GBAA4043, and GBAA0053) belonging to this GO functional term encode the sigma factors SigG and SigE, and the stage V sporulation protein T, respectively. The upregulation of sporulation-related genes may suggest that the Δ*htrA* strain-initiated sporulation process in response to its inability to cope with the oxidative stress.

### 3.8. Specificity of Selected Genes to the Response to Oxidative Stress

**Figure 5:**
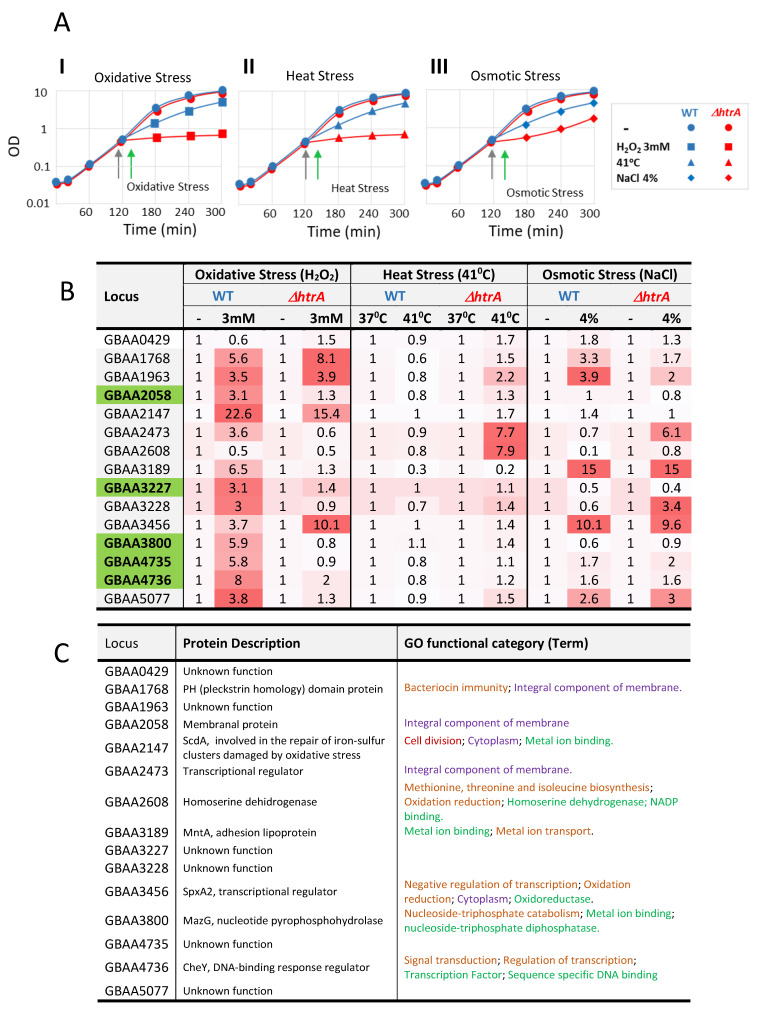
RT-PCR analysis of the transcription level of 15 Class II genes following oxidative, heat, and osmotic stress. (**A**) Growth profile of the WT (blue curves) and Δ*htrA* (red curves) strains in the presence or absence of 3 mM H_2_O_2_ (graph I), 41 °C (graph II), or 4%NaCl (graph III). Growth curves in the absence of stress at 37 °C are indicated by circles, in the presence of H_2_O_2_ by squares, at 41 °C by triangles, and in the presence of NaCl by diamonds. Gray arrows indicate the time of culture splitting into treated and untreated groups. Green arrows indicate the time of cell collection for RNA preparation. (**B**) Fold-change of 15 individual genes (indicated by their NCBI locus tag, on the left column) following the 3 stress regimens in either one of the strains. The transcription level in the absence of stress regimen was considered as 1 and that following stress treatment indicate the fold-change. Red gradient backgrounds indicate the extent of upregulation. Green background of locus tags indicates genes upregulated only upon H_2_O_2_ treatment and only in the WT strain. (**C**) Functional annotations of the proteins encoded by the 15 selected genes. The colors of the GO functional categories indicate the GO division (root) to which they belong: biological processes (in brown), molecular function (green), and cellular component (violet).

We have previously shown that the Δ*htrA* strain exhibited increased sensitivity not only to oxidative stress, but also to heat and osmotic stress [44]. Genes whose pattern of expression are modified in response to oxidative stress may belong to regulons which are affected by other stress regimens as well (and thus represent a general stress response). As the present study aimed at identification of genes specifically involved in the response to oxidative stress in an HtrA-dependent manner, a group of selected genes (see below) was further interrogated by RT-PCR for their modulation following heat and osmotic stress. We hypothesized that the group of DEGs that in response to H_2_O_2_ are specifically modulated in the WT while exhibiting unaffected transcriptional levels in the Δ*htrA* strain (378 genes in Classes II and III, see Figure 3A,C,D; listed in Supplementary Table S3) represented the most relevant categories that may encode functions related to the HtrA-dependent susceptibility to oxidative stress. Increasing the induction or repression index to 5-fold, reduced the number of DEGs to 15 genes in each one of the two classes (Figure 3D). Fifteen genes (Tabulated in Figure 5B,C) that were uniquely upregulated in the WT strain (Class II) by at least 5-fold (upon induction of oxidative stress, Padj < 0.01) were selected for further analysis.

These genes encode for a variety of functions and therefore, belong to various GO categories (Figure 5C). The products of six of the selected genes (GBAA0429, GBAA1963, GBAA3227, GBAA3228, GBAA4735, and GBAA5077) did not exhibit any recognizable function, based on the BLAST analysis carried out for functional assignment of the ORFs of the *B. anthracis* genome, against all bacterial genomes available in the NCBI or the EMBL databases. Upregulation of the transcription of these genes under stress conditions, as evidenced in the present study, may be beneficial for future elucidation of their biological function. Of note, the loci GBAA1963 and GBAA4735 were annotated (in the Ensemble website of the European Bioinformatic Institute-EMI of EMBL, http://www.ensemble.org) as pseudogenes, based on the alleged presence of signals for premature translation termination. Yet, our present observations demonstrating their expression (at least at the transcriptional level) clearly imply that these ORFs represent bona fide genes.

The analysis of the 15 selected class II genes included evaluation (using RT-PCR) of their expression level upon induction of heat, osmotic, and oxidative stress (Figure 5). The results of the real-time PCR analysis following H_2_O_2_ treatment (Figure 5B) were comparable to those of the RNA-seq, based on the observation that 13 out of the 15 genes exhibited upregulation of more than 3-fold in the WT strain. Nine genes demonstrated over 3-fold differential induction only in the WT strain following induction of oxidative stress. Interestingly, none of the genes that were induced in the WT upon H_2_O_2_ treatment were induced upon induction of heat stress (Figure 5B). These results suggested that the two stress regimens activate different regulons. Two genes (GBAA2473 and GBAA2608) exhibited transcriptional increase in the mutant strain only upon heat stress, implying that they may encode HtrA compensatory functions not specific to oxidative stress. Of note, one of these genes (GBAA2473) was induced in the mutant strain upon salt stress as well.

Seven genes (GBAA1768, GBAA1963, GBAA2473, GBAA3189, GBAA3228, GBAA3456, and GBAA5077) exhibited induction also upon salt stress and therefore are not oxidative stress-specific. Interestingly, one of these genes, GBAA3189, encodes for the MntA protein, an Mn^2+^ ABC transporter which was extensively studied in the past in our laboratory by mutagenesis studies [18]. MntA was demonstrated to represent a novel *B. anthracis* virulence determinant, and its role in *B. anthracis* pathogenesis was attributed to involvement in response to oxidative stress. Yet, the current study suggests that MntA may be involved also in the response to other environmental insults such as osmotic stress. In summary, 5 out of the 15 inspected upregulated genes (GBAA2058, GBAA3227, GBAA3800, GBAA4735, and GBAA4736) exhibited H_2_O_2_ specific induction in the WT.

### 3.9. Preliminary Evaluation of the Involvement of the Gene Encoding for MazG (GBAA3800) in Oxidative Stress Response

To further confirm that the functional screening distinguished genes potentially involved in the response to oxidative stress, one of the genes, *mazG* (GBAA3800), was selected as a first target for individual inspection. The data in Figure 5 clearly demonstrated that the expression of *mazG* was significantly upregulated (over 5-fold) upon peroxide treatment only in the parental WT strain. Furthermore, its expression appeared to be upregulated only in response to peroxide but not to other stress regimens. The gene GBAA3800 encodes for MazG, an NTP pyrophophyhdyrolase belonging to a toxin–antitoxin bacterial system [105,106], which has been shown to play a role in the oxidative stress response in *Mycobacterium smegmatis* [107].

**Figure 6:**
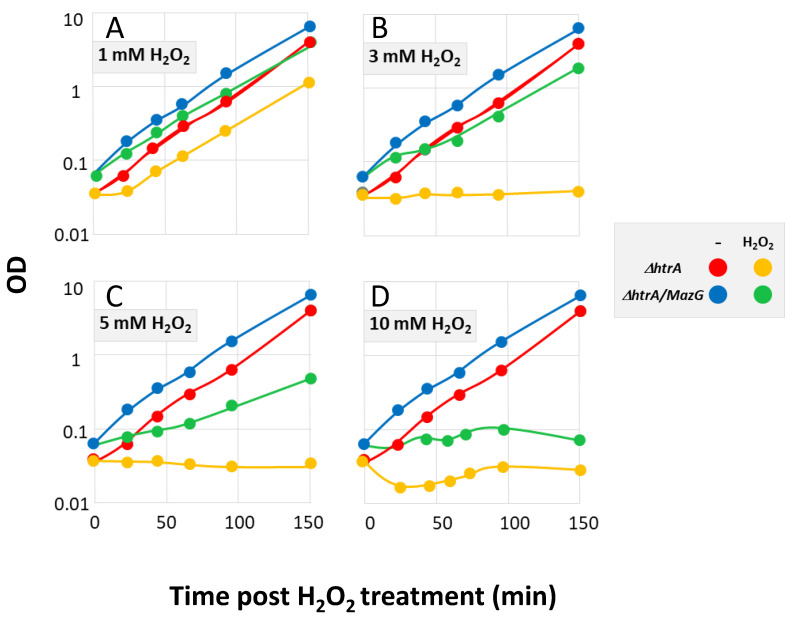
Growth of a MazG overexpressing Δ*htrA*-strain under oxidative stress. *B. anthracis* cells of the Δ*htrA* strain or Δ*htrA* overexpressing MazG (referred as Δ*htrA*/MazG) were grown in the absence (red circles: Δ*htrA*; blue circles: Δ*htrA*/MazG) or presence (yellow circles: Δ*htrA*; green circles: Δ*htrA*/MazG) of H_2_O_2_, as indicated in the legend on the right side. Panels (**A**–**D**) represent increasing concentrations of H_2_O_2_, as indicated above the curves. The growth curves in the absence of stress regimen are included in each panel for clarity. The curves describe the growth after initiation of the H_2_O_2_ treatment (considered time 0) of a representative experiment. Two additional experiments were carried out yielding similar results.

The gene *mazG* was cloned and overexpressed in Δ*htrA* strains derived from both the non-toxinogenic ΔVollum and the toxinogenic Sterne strains. The data in Figure 6A–D showed that the Δ*htrA* strain, as anticipated, is unable to withstand oxidative stress, revealing a growth arrest in the presence of H_2_O_2_ at a concentration as low as 1 mM H_2_O_2_. In contrast, MazG overexpression in the Δ*htrA* strain resulted in full resistance to 1 and 3 mM H_2_O_2_ and partial resistance to 5 mM H_2_O_2_. Increasing the oxidative stress to 10 mM H_2_O_2_ resulted in complete growth arrest. Of note, we did not detect any change in the virulence of a SterneΔ*htrA* strain overexpressing MazG in a murine model of anthrax infection (data not shown), which is consistent with the observation that abrogation of HtrA resulted in the modification of expression of hundreds of genes. Yet, the observation that MazG overexpression enhanced the resilience to H_2_O_2_ treatment confirmed that the reductional screen documented in the current report enabled identification of factors involved in the response to oxidative stress.

In conclusion, the study suggests that in *B. anthracis*, HtrA acts as a pleiotropic master factor of the response to stress, influencing the expression modulation of over 1000 genes (almost one-fifth of *B. anthracis* ORFs) under stress conditions. Future studies based on co-expression of several genes selected from the current screen may enable further elucidation of the role of HtrA-dependent novel functions (other than the classic toxins) in manifestation of *B. anthracis* virulence.

## Supplementary Materials

The following are available online at https://www.mdpi.com/2076-2607/8/12/1896/s1, Table S1: Oligo-DNA primers employed for RT-PCR analysis, Table S2: Differentially expressed genes following H_2_O_2_ treatment in both strains, Table S3: Differentially expressed genes following H_2_O_2_ treatment in the WT strain. Sheet 1-Upregulated genes; Sheet 2-Downregulated genes, Table S4: Differentially expressed genes following H_2_O_2_ treatment in the Δ*htrA* strain strain. Sheet 1-Upregulated genes; Sheet 2-Downregulated genes.
