# Supplementary material for: Comparative Analysis of the Global Transcriptomic Response to Oxidative Stress of Bacillus anthracis htrA-Disrupted and Parental Wild Type Strains
Source: Microorganisms. 2020 Nov 30;8(12):1896. doi: 10.3390/microorganisms8121896 (PMC7760947; doi:10.3390/microorganisms8121896)
Supplement: Supplementary file 1 [file microorganisms-08-01896-s001.zip › Supplementary Table S1.pdf]

Table 1. Oligo-DNA primers employed for RT-PCR analysis

Locus tag/*gene*	Forward/Reverse	Sequence (5'->3')
GBAA3660 *htrA*	F	AAACGAGAGAAGTGAGAAAATCGG
	R	TGGTGCCGAAAACTAATTG
GBAA5290 *dps2*	F	CCATTGGTACGTAAAAGGACC
	R	ATGGTTTGCCGCCAATTGC
GBAA1159 *katB*	F	ATCAAAACCCATCTACAATTAA
	R	ATCGCGGTAAGCTGGGTCTTC
GBAA0345 *ahpC*	F	CTATCGGTAAAATCGAGTACA
	R	GCAAGACCTTCTTCTTCCATTA
GBAA0429	F	CAAGGTGGCGAAGTAGTTGAATC
	R	AGTATGCATTTAGTTTTGGCAGTGA
GBAA1768	F	AATGGACTAGACAGCAATTAGAAATC
	R	GCGGACAGTTTTCTTTTAAGACTGA
GBAA1963	F	GAAGCAAAACGGATTCATCATG
	R	CGAACGATTAATAACAGCATAACTATC
GBAA2058	F	AACGTGTCGGAATGATTTCAATC
	R	GCGCTTTTTTATGCATTAATTCG
GBAA2147 *scdA*	F	AACCACGCTGGCAACATTATT
	R	GGCGATTGTAAACAAGTCGATAAGT
GBAA2473	F	GAGGTCATATGTAACAGAAGGGTTTG
	R	TGTGGTATCTTCTTTTGGAGATGGT
GBAA2608	F	ACAGTTTAATGGGAACAGAGAATAAG
	R	TTATTTTGTTCCTTCGCATTTCG
GBAA3189 *mntA*	F	CGCATGAATATGATCCACTACCA
	R	AACCACGCTCCACCTTCTTCT
GBAA3227	F	TGACAGCTTAACACAATCTACATTTGA
	R	TATTACGCCTGTTCTAGCACTAACTTT
GBAA3228	F	AAAACACTACCTGCTGCGAATGA
	R	CTCCAACTGGTTTCGTGTTATTTG
GBAA3456 *spxA2*	F	TTCATGTCGAAAAGCGAAAGC
	R	GGACTTAAGTTCATCCACTGTCATAGA
GBAA3800 *mazG*	F	GCATCTTCACTACTTTTCCACTGAAA
	R	CGAGATGATAGAGACTGGAAGCAA
GBAA4735	F	GTTGCAACAGTCTTTATATCATCAACT
	R	TCTTTGCAAACGGGCTAAGC
GBAA4736 *CheY*	F	CACCCACATTGGTGCATCTC
	R	TCGGAGTCATTCCAGTCATTTTAC
GBAA5077	F	AAAGCAATAAACAAGTGGGAACGT
	R	CGAATTTTGCTGCTTCATCCA
GBAA4533 *gatB*	F	AGCTGGTCGTGAAGACCTTG
	R	CGGCATAACAGCAGTCATCA
